# Data Resource Profile: Results Analysis Base of Navarre (BARDENA)

**DOI:** 10.1093/ije/dyad144

**Published:** 2023-10-28

**Authors:** Javier Gorricho, Leire Leache, Ibai Tamayo, Francisco Sánchez-Sáez, Maite Almirantearena, Edurne San Román, Jerónimo Ballaz, Javier Turumbay, Julián Librero

**Affiliations:** Servicio de Evaluación y Difusión de Resultados en Salud, Servicio Navarro de Salud-Osasunbidea, Pamplona, Spain; IdiSNA, Instituto de Investigación Sanitaria de Navarra, Pamplona, Spain; IdiSNA, Instituto de Investigación Sanitaria de Navarra, Pamplona, Spain; Sección de Innovación y Organización, Servicio Navarro de Salud-Osasunbidea, Pamplona, Spain; IdiSNA, Instituto de Investigación Sanitaria de Navarra, Pamplona, Spain; Unidad de Metodología-Navarrabiomed, Hospital Universitario de Navarra (HUN), Universidad Pública de Navarra (UPNA), Pamplona, Spain; Red de Investigación en Cronicidad, Atención Primaria y Promoción de la Salud (RICAPPS), Spain; School of Engineering and Technology, Universidad Internacional de La Rioja, Logroño, Spain; Servicio de Evaluación y Difusión de Resultados en Salud, Servicio Navarro de Salud-Osasunbidea, Pamplona, Spain; Servicio de Tecnologías de Salud, Dirección General de Telecomunicaciones y Digitalización, Pamplona, Spain; Servicio de Tecnologías de Salud, Dirección General de Telecomunicaciones y Digitalización, Pamplona, Spain; Subdirección de Sistemas y Tecnologías para la Salud, Servicio Navarro de Salud-Osasunbidea, Pamplona, Spain; Unidad de Metodología-Navarrabiomed, Hospital Universitario de Navarra (HUN), Universidad Pública de Navarra (UPNA), Pamplona, Spain; Red de Investigación en Cronicidad, Atención Primaria y Promoción de la Salud (RICAPPS), Spain

**Keywords:** BARDENA, Results Analysis Base of Navarre, health care, data warehouse, population data

Key Features

- The Results Analysis Base of Navarre (BARDENA) is a population data warehouse of the Navarre Health Department (Spain), which includes weekly updated information since 2012.
- BARDENA integrates individual-level information generated at any level of health care of approximately 97% of the Navarre population, totalling more than 660 000 people.
- Information stored in BARDENA Core can be accessed by health care decision makers and researchers upon approval of applications by the BARDENA Data Committee.
- BARDENA is committed to improving quality of health care, facilitating decision making and promoting research.
- BARDENA interoperates with national and international databases, and has been used in multiple research projects and studies.

## Data resource basics

### Spanish National Health System (NHS)

Spain has a decentralized health system that operates as a network of 17 regional health services. It is publicly funded (mainly from taxes), and provides universal, free of charge, needs-based care coverage to the resident population of Spain. The Ministry of Health is responsible for the national health planning, coordination and regulation, but other competences such as the primary jurisdiction over-operational planning, resource acquisition, allocation and provision are devolved to the regional health authorities.^1^ In practice the regional health services, such as the Navarre Health Service, are organized into Health Departments (each one grouping several primary health care districts of more than 5000–25 000 people each) and are responsible for providing global health access to their reference population in primary, hospital and specialized outpatient care.

However, it is worth noting that whereas the health assistance is free of charge, medicines and medical devices (including orthotics and prosthetics such as wheelchairs, hearing aids etc.) are subject to a co-payment,^1^ which is established on the basis of the economic income and employment status of each individual.

### The Spanish region of Navarre

Navarre is a region in the north of Spain with more than 660 000 inhabitants, representing 1.4% of the Spanish population, and approximately 1.5/1000 of the European population. The mean age of the Navarre population is 43.8 years, slightly lower than the overall mean age in Spain (44.1 years). The age distribution in Navarre is the following: ≤19 years: 20%; 20–39 years: 22%; 40–59 years: 31%; 60–79 years: 20%; and ≥80 years: 6%. The annual birth rate is around 7/1000 inhabitants (4765 births in 2022).^2^

**Figure 1.**
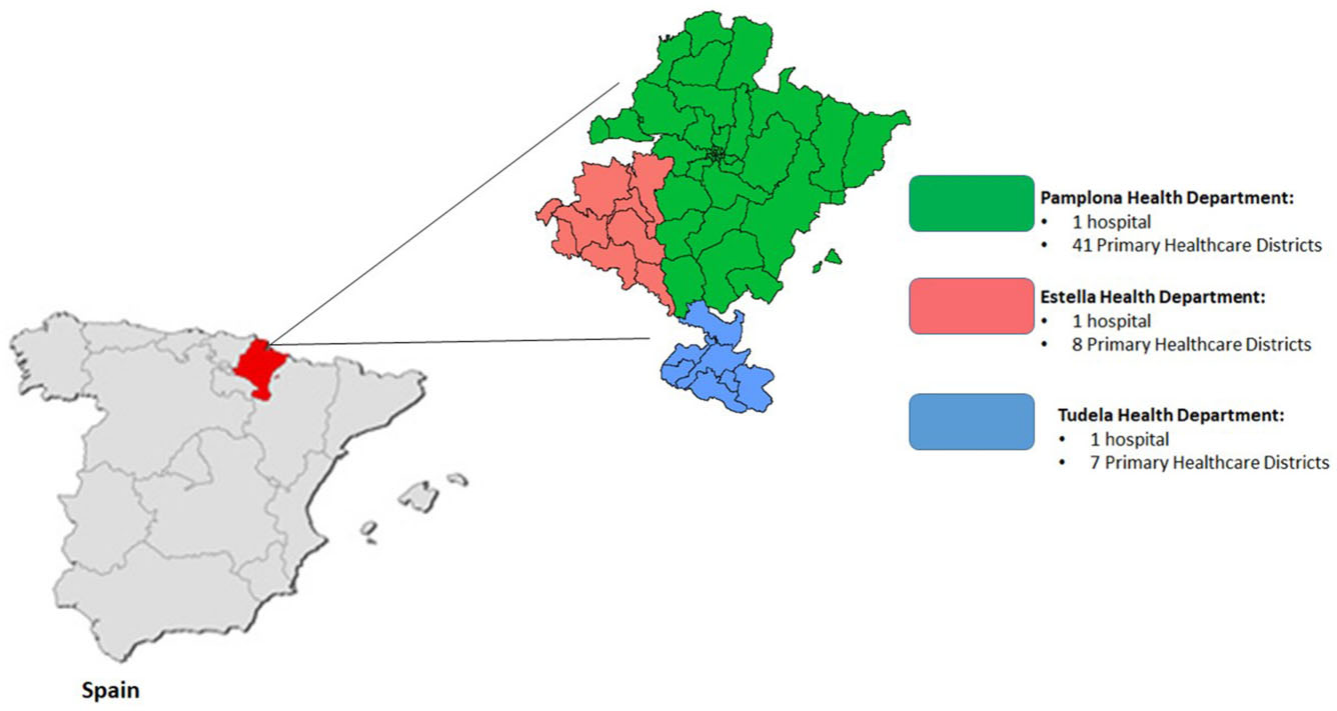
Geographical location of Navarre and administrative organization of the Navarre Health Service

Health care for the population of Navarre is provided through the publicly financed Navarre Health Service, which includes three Health Departments and 56 Primary Health Care Districts. The organization of the Navarre Health Service is represented in Figure 1.

### Results Analysis Base of Navarre (BARDENA)

Each regional health service holds the health information of its reference population, and therefore develops and operates its own information systems. Navarre is no exception, and here is where the Results Analysis Base of Navarre (BARDENA) comes into play by driving the integration of Navarre’s health information systems.

BARDENA is a publicly funded data warehouse, property of the Department of Health of the Government of Navarre based in Pamplona. It is managed by administrators of the Navarre Health Service, who are responsible not only for its operation but also for the validity and quality of the information. It was created in 2015 in order to audit processes, evaluate health results, generate reports for the different health care decision makers and promote health research.

As such, BARDENA sequentially includes health-related information generated by the Navarre Health Service for the population of the region, regardless of the area or setting from which the health care attention has been provided. At this moment, BARDENA includes health records of more than 600 000 people, approximately 97% of the Navarre population.

The data warehouse includes exhaustive longitudinal patient-level information, providing a wide follow-up of the population, which increases over time. This is done through the integration of all the information generated from each person’s first contact with the system (usually from birth) until death, and without any time limit.

## Data collected

### Structure and data included in BARDENA

As stated before, BARDENA is continuously supplied from different data sources, which together constitute the BARDENA Core, which is the backbone of the data warehouse. BARDENA Core is made up of five dimensions: person, location, centre, professional, and clinical and other information.

The ‘person’ dimension includes administrative and sociodemographic data. The ‘location’ dimension comprises data on geographical location of the residence and the primary care centre of reference; and the ‘centre’ dimension includes the identification of the primary care centre of reference. The ‘professional’ dimension includes information on the health care professional of reference. The ‘clinical and other information’ dimension includes lifestyle and economic data, information on preventive and diagnostic procedures, medical diagnoses, medical and nursing interventions, pharmaceutical data, social assistance and information on contact with health services. Table 1 describes the dimensions, categories and data included in BARDENA.

**Table 1.** Dimensions, categories and data included in BARDENA

Dimension	Category	Data included
Person	Administrative data	Health insurance, date of birth, date of first contact with the Navarre Health Service, date of death
	Sociodemographic data	Sex, country of birth, nationality, employment status, risk of social exclusion, address
Location	Geographical location	Geolocation of the residence and the primary care centre of reference
Centre	Health care unit	Primary care centre of reference
Professional	Health care professional	Physician of reference, profile of the professional
Clinical and other information	Lifestyle data	Smoking, alcohol intake, physical activity
	Economic data (analytical accountability)	Pharmaceutical co-payment for each individual according to income level Unit cost of the human^a^ and material resources (drugs, medical devices etc) employed in the surgical and non-surgical interventions carried out in the Navarre Health Service
	Preventive procedures	Vaccination (type of vaccine, manufacturer, batch number, number of doses, administration date and location, adverse reactions related to vaccines, rejected vaccinations and, if applicable, risk groups)
	Diagnostic procedures	Biochemical/laboratory, microbiological, anatomical pathology, radiological data
	Medical diagnoses	Medical diagnoses registered at any level of care
	Medical interventions	Surgical and non-surgical medical procedures
	Nursing interventions and measurements	Blood pressure, pulse, heart rate, respiratory rate, oxygen saturation, weight, height, body mass index
	Pharmaceutical data	Drug prescription (prescription date, treatment duration, drug prescribed, dose, prescriber etc) in both inpatient and outpatient settings Drug dispensing to outpatients (number of packages dispensed, date of dispensation etc)
	Social assistance	Interventions conducted by social workers (e.g. providing support to patients and caregivers, assisting patients in health care transitions, managing social health centres, processing applications for financial and social assistance, and assessment of patients’ functional capacity). Type of intervention done, the result of the intervention and beginning and end of the intervention are registered
	Contact with health services	Contact with hospitals, emergency services, specialized care centres (including mental and obstetric care, among others), primary care centres, public social care centres and other services provided by the Navarre Health Service (such as palliative care and home hospitalization units). Both visits and admissions (including the date of admission and discharge) are registered

a The cost of the human resources is estimated through salaries.

### Sources of data

**Figure 2.**
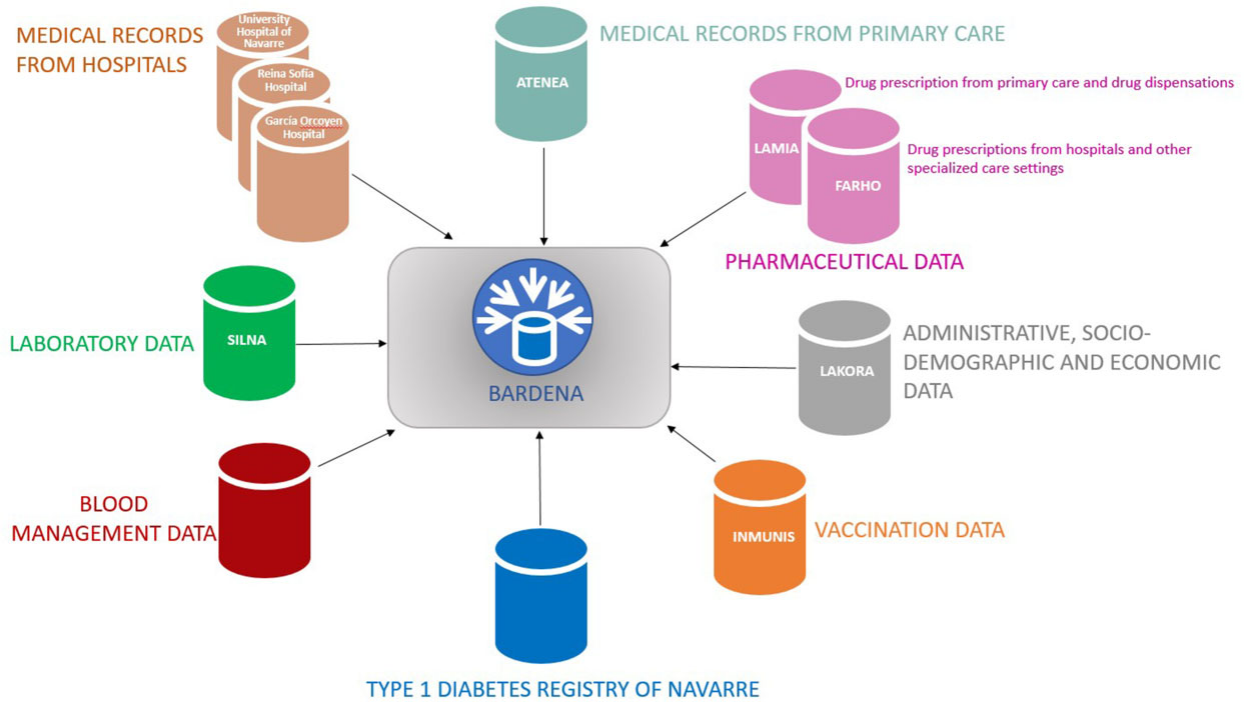
BARDENA data sources

The different sources of information from which BARDENA draws are shown in Figure 2 and described below.

The Population Information System (LAKORA) is the module that provides administrative, sociodemographic and economic data. LAKORA is the source of the unique and permanent pseudonymized personal identifier associated with each individual.

The module of primary care information (ATENEA), implemented in 2003, includes the electronic medical records of primary care, reaching a 95% coverage of the population in 2008. ATENEA includes information on both paediatric and adult primary care as well as any related social assistance and nursing care. Diagnoses in ATENEA are coded through the second edition of the International Classification of Primary Care (ICPC-2).^3^

The pharmaceutical data are registered in two modules according to the setting: LAMIA and FARHO. LAMIA includes drug prescriptions from primary care in outpatients and drugs dispensations in community pharmacies. FARHO includes drug prescriptions from hospitals and other specialized care settings in inpatients and outpatients. Both of them use the Anatomical Therapeutic Chemical (ATC) classification system^4^ and the (Spanish) National Pharmaceutical Catalog for drug prescription, allowing the identification of the exact content of each drug prescription and dispensation. LAMIA includes a comprehensive e-prescription system connected to all community pharmacies in Spain. This system links each drug prescription to the corresponding dispensation by the community pharmacy through a specific prescription identification number.

The Hospital Medical Records module was created in 2001 and provides comprehensive information covering all areas of specialized care. It includes data on hospital admissions (both in-hospital and home hospitalizations), specialized care consultations (including mental and prenatal care, among others), emergencies, diagnostic procedures, medical and nursing procedures, surgeries, prevention and safety measures, and interventions conducted by social workers in inpatients.

Information on medical diagnoses and procedures included in the Hospital Medical Records module are obtained from the Minimum Basic Data Set at Hospital Discharge (MBDS). This is a synopsis of clinical and administrative information on all hospital admissions and major outpatient surgery which all hospitals from the whole NHS are mandatorily required to complete. Since its implementation in 1987, information from public and private hospitals has been progressively incorporated. To date, MBDS is the largest administrative and clinical database available in the Spanish NHS.^5^

MBDS includes information on age, sex, geographical area, hospital name, date of hospital admission and discharge, principal and secondary diagnoses at hospital discharge, diagnostic and therapeutic procedures conducted during the hospital stay, complications, in-hospital mortality and destination at discharge. In each hospital, the MBDS database is completed by trained coding staff based on the medical discharge report and other information available in the clinical records.^5^ Medical diagnoses and procedures are coded in MBDS through the International Statistical Classification of Diseases (ICD).^6^ The ninth version of the ICD (ICD-9) was used until December 2015 and the tenth version (ICD-10) was adopted thereafter.

The SILNA module includes laboratory data. There is also a specific module that includes information related to blood management (blood transfusions, donations etc.).

The INMUNIS module stores all the information on vaccination in Navarre since 2000. Available data include the type of vaccine, manufacturer, batch number, number of doses, administration date and location, adverse reactions related to vaccines, rejected vaccinations and, if applicable, risk groups. Data on COVID-19 vaccination are also included.

BARDENA also includes information from specific registries, such as the type 1 diabetes registry of Navarre, created as part of the good practices initiative of the Spanish NHS.^7^

### Unique identifier

BARDENA contains individual-level information. LAKORA is the source of the unique and permanent pseudonymized identifier associated to each individual, which guarantees the confidentiality of the information. This identifier is shared between the different modules of information that supply BARDENA, allowing data linkage across the multiple databases and ensuring the traceability of the individual patient data.

### Data integration and validation

BARDENA data collection started in 2012, but gathered all historical data from the existing information systems in the Navarre Health Service (hospitalizations and clinical diagnosis since 1996). The information generated as routine practice is integrated in BARDENA on a weekly basis. In the case of MBDS, data are subject to validation and consolidation processes before their integration in BARDENA. During these processes, quality reports are automatically generated, which are revised and managed by the administrators of the database in order to detect and correct inconsistencies. When inconsistencies or incorrect values are detected during the validation processes, these are flagged and returned to the module of origin for revision before including them in BARDENA. Therefore in this case, data from the last quarter before the data extraction may be missing or non-consolidated in BARDENA.

BARDENA is hosted on local servers using SQL Server 2016. Specifically, BARDENA makes use of the SQL Server Integration Service for the Extract, Transform and Load (ETL) processes.

## Data resource use

### Experience with the use of BARDENA in research projects and studies

BARDENA has been used for multiple national and international research projects and studies on various clinical and epidemiological issues.^8–27^Supplementary Table S1 (available as Supplementary data at *IJE* online) includes details of these studies.

BARDENA also contributes with information from the hospital setting to Spanish national networks, such as the Atlas of Variations in Medical Practice in the Spanish NHS.^28–34^ This is an initiative aimed at promoting equity, quality and efficiency of the NHS through the analysis of systematic and unwarranted geographical variations in access to hospital resources and in medical practice.^31^

### BARDENA interoperability with national and international databases

Individual patient data corresponding to hospital health care included in BARDENA (demographics, medical diagnoses, procedures, in-hospital mortality etc.) are transmitted to the Spanish Ministry of Health. This information is integrated with the data of the rest of the Spanish regions in the MBDS database, which is the database of reference of the Spanish NHS.^5^ This allows for interoperability of the information and analysis of variability between the Spanish hospitals.

In addition, BARDENA information is integrated in the Spanish database for the Pharmacoepidemiological Research Database for Public Health System (BIFAP), managed by the Spanish Agency of Medicines and Medical Devices. This database was created in 2001 and at this moment includes more than 20 million medical records.^35^ Work is also under way to integrate information from BARDENA into the Spanish Health Data lake that will be created in the near future.^36^

Moreover, at present BARDENA is in the process of being integrated into the European Health Data & Evidence Network (EHDEN), a consortium of data sources from 12 countries, funded by the European Union’s Horizon 2020.^37^ EHDEN was launched in 2018 and aimed at building a standardized large-scale network to reduce the time needed to provide answers in real-world health research. This involves transforming data into the Observational Medical Outcomes Partnership (OMOP) Common Data Model (CDM),^38^ an open community data standard designed to standardize the structure of the data from the different sources. This system allows other organizations to analyse and consult our data warehouse through the Observational Health Data Sciences and Informatics (OHDSI) program.^39^

## Strengths and weaknesses

BARDENA has several strengths and some differential features with regard to other information resources. First, it links population-wide individual data including administrative, sociodemographic, geographical, economic and clinical information, lifestyle data etc. This allows for the study of the impact of social determinants of health (including age, sex, ethnicity and income level) on the access and use of health resources and also on clinical outcomes at individual level in the Navarre population.

Second, BARDENA includes information from almost all the Navarre population, being therefore representative. This makes it possible to carry out observational studies in specific subpopulations excluded from clinical trials, such as people from ethnic minorities, elderly and paediatric populations,^18^^,^^26^ people with multiple chronic diseases,^25^ cancer patients,^24^ people with polypharmacy and pregnant women, among others, with a high precision.

Third, information in BARDENA is updated on a weekly basis, making it available in a short period of time. This facilitates decision making in situations that require an agile response, such as in the COVID-19 pandemic, and also makes it possible to forecast material and human resources that will be needed in the near future.

Fourth, BARDENA allows for the construction and follow-up of large cohorts of patients over time and the development of longitudinal studies, enabling monitoring of health outcomes in the long term.

Fifth, data quality in most of the information modules is distinctively high, providing insight into a population of more than 600 000 inhabitants.

Sixth, the availability of unit cost data per patient and activity allows for economic evaluation studies with high specificity and granularity.

Seventh, as a source of population health records, BARDENA can contribute to pragmatic studies by providing a sample of patients undergoing routine practice. This reduces the cost and timing of access to data compared with studies with experimental designs, such clinical trials.

Eighth, BARDENA is an ultimate tool for pharmacoepidemiology, which integrates linked information on clinical conditions, drug prescriptions and dispensations at the individual level, enabling accurate drug utilization and adherence studies.

Ninth, BARDENA Core can interact with other integrated tools and information systems beyond health issues, such as those from education, social affairs and government transparency sites.

Tenth, information stored in BARDENA Core can be easily visualized by health care decision makers and researchers via the Tableau server, which provides statistics of aggregated data, tables and figures.

Eleventh, BARDENA allows for data mining, which can be used for identifying clinical processes that permit the application of improved protocols, monitoring the performance of current processes, detecting and removing bottlenecks, accelerating clinical processes and simulating how the introduction of changes in the processes may affect the system.

Twelfth, BARDENA interoperates with national and European databases, promoting the development of knowledge and population research at a macro level. Finally, the verification and quality processes to which BARDENA is subjected, guarantee the validity of the information. In this regard, a high effort is being made to integrate and audit the information, resulting in high-quality information that strengthens data exploitation capabilities.

BARDENA also has some limitations. Some of the information modules that comprise BARDENA Core are subject to the inherent constraints of the routine clinical practice electronic databases. In some specific processes, information bias may occur due to absent registration (data incompleteness), registration delay (e.g. in cases of acute stroke or cardiorespiratory arrest) or differing data recording practices (data inaccuracies, misclassification and heterogeneity), although these are intrinsic problems of any repository using data from routine clinical practice. In addition, information modules were created at different time points, and therefore cover different periods. Moreover, there is a lack of data on specific mortality causes and on drug prescriptions in patients from private nursing homes. However, pharmacological information from private nursing homes is being progressively integrated into BARDENA and is expected to be completed in the forthcoming years. Finally, BARDENA does not include information on people who do not receive assistance from the Navarre Health Service, that is who are attended exclusively at private centres. However, this is an unusual situation in Navarre, estimated at approximately 3% of the population.

## Data resource access

### Ownership, management and access to BARDENA

BARDENA is owned by the Health Department of the Government of Navarre and operated by managers from the Navarre Health Service. Therefore, the Navarre Health Service serves as custodian for BARDENA, ensuring the proper management, safeguarding and responsible use of the data. BARDENA information can be used for improving quality of health care and decision making and also for research purposes. Data are currently available for health care decision makers and researchers (internal and external to the Navarre Health Service). Industry-funded studies are not accepted. Access to data is free of charge.

Access to BARDENA information for research purposes requires a formal application accompanied by: (i) a complete study protocol that details the planned purpose of the use of data; (ii) the approval of the study protocol by an accredited ethical research committee; and (iii) the informed consent of patients or a waiver granted by an ethics committee.

BARDENA has a committee (BARDENA Data Committee) that revises the applications for access to data from BARDENA, approves or denies access to it and establishes priorities. The applications for access must be submitted electronically to the Management Office of the BARDENA Data Committee [salsedrs@navarra.es]. Following authorization, researchers must sign a document in which they commit themselves to keep the data in a secure environment, not to attempt to re-identify patients or cross data with other databases, not to use the data for purposes or projects other than those specified in the study protocol (including commercial purposes) and not to transfer data to third parties. Data provided will be those strictly necessary for the study conduct. In case researchers claim for information additional to that in the original protocol, a new application must be submitted. The above-mentioned commitments limit the possibility of sharing raw data in open repositories or publishing individual patient data.

### Data visualization and data mining

The Health Department of the Government of Navarre ensures the pseudonymization of the data extracted from BARDENA by providing to the applicant (health care decision makers or researchers) the de-identified datasets. Aggregated data from BARDENA Core are also made available to health care decision makers and researchers via the Tableau server.^40^ This BARDENA diffusion product allows for production of analysis tables and visuals of the main information from BARDENA Core.

BARDENA Milenia is the specific tool that allows BARDENA Suite to perform data mining. These data analyses are carried out using software such as R, Python or ProM, providing BARDENA with advanced functionality.

## Ethics approval

Not applicable.

## Supplementary Material

dyad144_Supplementary_Data

## Data Availability

BARDENA data are accessible under the conditions described in Data Resource Access, above.
