# Supplementary material for: Data Resource Profile: Results Analysis Base of Navarre (BARDENA)
Source: Int J Epidemiol. 2023 Oct 28;52(6):e301–7. doi: 10.1093/ije/dyad144 (PMC10749752; doi:10.1093/ije/dyad144)
Supplement: dyad144_Supplementary_Data [file dyad144_supplementary_data.docx]

**Supplementary data**

**Table S1.** Studies conducted with information from BARDENA

| **Thematic area** | **Specific topic** | **Subject analyzed and main findings** | **Information modules from BARDENA used** |
| --- | --- | --- | --- |
| Infectious diseases | COVID-19 | - **COVID-19 VE**:   -COVID-19 VE was moderate in preventing infection and was higher against symptomatic and hospitalized cases.^8^  -Vaccines provide good protection against COVID-19 infection among adults ≥ 65 years, particularly among fully vaccinated individuals.^9^  -Two doses were highly effective against hospitalization in close contacts, but suboptimal for infection control.^10^  -COVID-19 VE against symptomatic infection was 65% at ≥90 days between vaccination and onset.^11^   - **COVID-19 and ABO group**:   -0 blood group has a lower risk of infection and A blood group has the highest. There were no differences in hospitalization, ICU or death.^12^  -There were no relevant differences between the overall adult population and population aged ≥60 years in the risk of COVID‐19 infection and severity according to ABO groups.^13^   - **Risk factors for COVID-19 infection, hospitalization and death**: the study supports the prioritization of the older population, nursing home residents, and people with chronic conditions and functional dependence for SARS-CoV-2 prevention and vaccination^14^ - **COVID-19 and hypertension**: Hypertension was not independently associated with a higher risk of nor severe COVID-19.^15^ - **COVID-19 and obesity**: severe obesity is a relevant risk factor for COVID-19 hospitalization and severity in young adults.^16^ | Modules of medical records from hospitals, ATENEA, LAKORA, SILNA, INMUNIS, module that contains blood management data |
|  | HCV infection | Progress in the elimination of hepatitis C virus infection: the prevalence of diagnosed active HCV infection has dropped by almost half over three years.^17^ | Modules of medical records from hospitals, ATENEA, LAKORA, SILNA |
| Mental health | ADHD | Trend and factors involved in the incidence of ADHD diagnoses in Navarre: there was a decreasing trend since 2015, with a clear period and age effect.^18^ | Modules of medical records from hospitals, ATENEA, LAKORA |
| Cardiovascular diseases | Diabetes | - **Effects of physical activity:**   -A tailored exercise intervention in hospitalized elderly diabetic patients was safe and effective for the prevention of functional and cognitive decline.^19^  -Physically active patients with type 2 diabetes may have a reduced risk of cardiovascular events.^20^   - **CV risk:**   -During 5 years, 0.8% patients with type 1 diabetes and 7.7% with type 2 developed a cardiovascular event.^21^  -Women showed worse control of CV risk factors.^22^   - **Impact of socioeconomic inequalities:**   -Socioeconomic inequalities in the achievement of cardiovascular and metabolic control in type 2 diabetic patients have been found.^23^ | Modules of medical records from hospitals, ATENEA, LAKORA, LAMIA, FHARO, SILNA, data from type 1 diabetes registry of Navarre |
| Cancer | Breast cancer | Use of health resources: higher use of health services in long-term breast cancer survivors than in women without breast cancer regardless of survival time.^24^ | Modules of medical records from hospitals, ATENEA, LAKORA, SILNA |
| Other chronic conditions | Non-cancer chronic conditions | - **Mortality prediction**: a mortality prediction model for patients with non-cancer multiple chronic conditions was developed and validated.^25^ | Modules of medical records from hospitals, ATENEA, LAKORA, LAMIA, FHARO, SILNA |
| Obesity | Childhood obesity | - **Selection bias in estimation of the prevalence of childhood obesity due to missing weight and height data from electronic health records:** weight status estimations are similar using complete data, multiple imputation and inverse-probability weighting.^26^ | Modules of medical records from hospitals, ATENEA, LAKORA |
| Safety | Adverse events during hospitalization | - **Need for redesign the safety policies implemented in hospitals based on avoiding 13 in the numbering of rooms/beds**: hospitals should pay attention to causes and interventions to avoid adverse events based on evidence rather than beliefs or myths.^27^ | Modules of medical records from hospitals, ATENEA, LAKORA |

ADHD: Attention Deficit Hyperactivity Disorder; CV: cardiovascular; HCV: hepatitis C virus; ICU: intensive care unit; VE: vaccine effectiveness
